# Nasal Polypus

**Published:** 1887-12

**Authors:** 


*Nasal Polypus.* By EDWARD WOAKES, M.D. (Lond.) Pp. 140. London: H. K. LEWIS. 1887.

A book of 140 pages on Nasal Polypus would seem to be the *ne plus ultra* of specialised medical writing. But this book treats of nasal polypus with neuralgia, hay-fever, and asthma in relation to ethmoiditis, and, as far as we can see, with pretty nearly every ill the flesh is heir to. As the author says in reference to nasal polypus, "it would appear difficult to limit the extent or variety of morbid phenomena which may originate in a comparatively small area." The nose is very properly regarded as the most important feature in the face; a perusal of this book has almost convinced us that it is the most important organ in the body. And so it probably is—to a man who has a polypus in it.

The subject is very well handled. Many original observations on the pathology and clinical bearings of "ethmoiditis" (as it is called), with its extensive reflex ramifications, are given. And if we venture to differ from some of the conclusions drawn as to the far-reaching influences which this disease has on the human frame, it is probably because we are not specialists on the nose.

